# The biological role of actinin-4 (*ACTN4*) in malignant phenotypes of cancer

**DOI:** 10.1186/s13578-015-0031-0

**Published:** 2015-08-18

**Authors:** Kazufumi Honda

**Affiliations:** Department of Chemotherapy and Clinical Research, National Cancer Center Research Institute, 5-1-1 Tsukiji Chuoku, Tokyo, 104-0045 Japan; AMED-CREST AMED, Japan Agency for Medical Research and Development, 1-7-1 Otemachi, Chiyoda, Tokyo, 100-0004 Japan

**Keywords:** Cancer invasion, Metastasis, Actinin-4 (ACTN4), Actin-bundling protein, Biomarker

## Abstract

Invasion and metastasis are malignant phenotypes in cancer that lead to patient death. Cell motility is involved in these processes. In 1998, we identified overexpression of the actin-bundling protein actinin-4 in several types of cancer. Protein expression of actinin-4 is closely associated with the invasive phenotypes of cancers. Actinin-4 is predominantly expressed in the cellular protrusions that stimulate the invasive phenotype in cancer cells and is essential for formation of cellular protrusions such as filopodia and lamellipodia. *ACTN4* (gene name encoding actinin-4 protein) is located on human chromosome 19q. *ACTN4* amplification is frequently observed in patients with carcinomas of the pancreas, ovary, lung, and salivary gland, and patients with *ACTN4* amplifications have worse outcomes than patients without amplification. In addition, nuclear distribution of actinin-4 is frequently observed in small cell lung, breast, and ovarian cancer. Actinin-4, when expressed in cancer cell nuclei, functions as a transcriptional co-activator. In this review, we summarize recent developments regarding the biological roles of actinin-4 in cancer invasion.

## Background

Despite successful complete resection at the primary cancer site, poor outcomes are occasionally observed in patients due to failure to control distant metastasis. Controlling metastasis is expected to improve the survival rate of patients with cancer [1, 2]. The mechanisms of cancer metastasis, which occurs in a multistep process, have been investigated to identify new therapeutic strategies for patients with cancer. During formation of metastatic lesions, carcinoma cells destroy the basement membrane, invade the surrounding extracellular matrix, intravasate through the endothelium into the circulation, extravasate again though the capillary vessels, and finally establish secondary tumors at distant sites [1, 3, 4]. The dynamic assembly of the actin cytoskeleton is important in this multistep process of forming metastatic lesions. In particular, the actin cytoskeleton plays important roles in the formation of cellular protrusions known as filopodia, lamellipodia, and invadopodia [5–10].

Alpha-actinin is an actin cross-linking protein that belongs to the spectrin superfamily. Four isoforms of alpha-actinin have been identified: alpha-actinin-1 (gene name; *ACTN1*) [11], actinin-2 (*ACTN2*) [12], actinin-3 (*ACTN3*) [12], and actinin-4 (*ACTN4*) [13]. These isoforms are classified into two groups: muscle (*ACTN2* and *ACTN3*) and non-muscle isoforms (*ACTN1* and *ACTN4*) [14]. Muscle-type isoforms of actinins are only expressed in skeletal and smooth muscle, where they mediate actin filament bundling and interactions with the Z-disk. On the other hand, non-muscle type isoforms are only expressed in non-muscle cells, where they also mediate actin filament bundling and interact with cell membranes. Non-muscle types in particular are associated with cell adhesion and cell migration. We originally identified *ACTN4* as a metastasis-related gene in cancer in 1998 [13] and have investigated the biological mechanisms and clinical implications of actinin-4 in cancer metastasis.

In this review, I mainly describe the involvement of actinin-4 in cancer metastasis and review recent studies of the biological function of actinin-4 in cancer and human diseases.

## Isolation of *ACTN4*, a metastasis-related gene

**Fig. 1:**
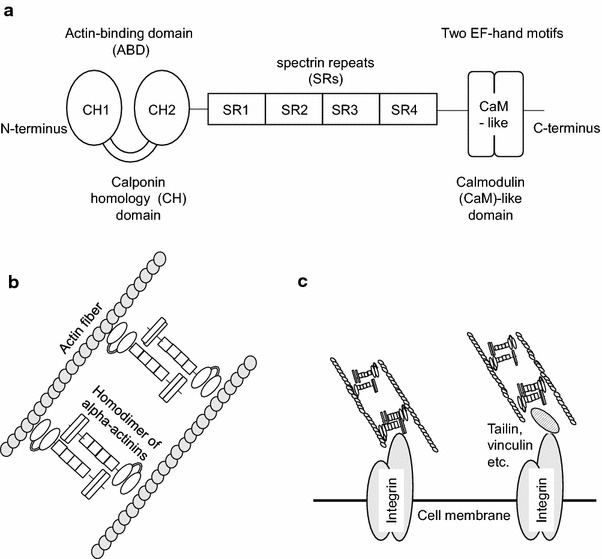
Schematic of the domain structure of alpha-actinins (**a**). Alpha-actinins are composed of an actin-binding domain (ABD), four spectrin repeats (SRs), and a calmodulin (CaM)-like domain. ABDs are composed of two calponin homology (CHs) domains (CH1 and CH2). CaM-like domains are composed of two EF-hand motifs. Schematic of actin bundling with alpha-actinins (**b**). Actinins form an anti-parallel dimer, and homodimers of actinins bundle actin fibers by interacting with the ABD. Schematic of interactions between actin fibers and cell membranes (**c**). Actin fibers bundled with actinins directly or indirectly interact with the cell membrane through integrins.

We generated a mouse monoclonal antibody that strongly reacts to the highly invasive phenotype of breast carcinoma, and we identified the full-length cDNA for the protein that was recognized by this antibody. This cDNA encodes a fourth novel isoform of alpha-actinin and was named actinin-4. Human actinin-4 is composed of 911 amino acids, and the amino acid homology with actinin-1 is 86% [13]. Alpha-actinin family members form an anti-parallel dimer with an actin-binding domain (ABD), which is composed of two calponin homology (CH) domains at the N-terminus of each monomer. Adjacent to the ABD, four spectrin repeats are followed by a C-terminal calmodulin (CaM)-like domain consisting of two EF-hand repeats (Fig. 1a) [14]. This molecular architecture results in the formation of a rod-shaped molecule with ABD and CaM domains at both ends, allowing cross-linking of bundles of actin filaments (Fig. 1b). Moreover, non-muscle alpha-actinins interact with actin filaments to connect with the plasma membrane through beta 1–3 integrins, vinculin, and alpha-catenin (Fig. 1c) [14–16].

Immunohistochemical analysis with the anti-actinin-4-specific antibody revealed significant overexpression of actinin-4 in histological subtypes of breast cancer with high invasive ability. Patients with overexpression of actinin-4 in invasive ductal adenocarcinoma of the breast show worse prognosis for overall survival than patients without overexpression. Immunocytochemistry following the wound healing assay to evaluate the invasiveness of cancer cells revealed that actinin-4 predominantly accumulates at artificial invasive fronts [13].

## The correlation between protein overexpression of actinin-4 and metastatic ability of cancer cells

Cancer cells, which are at the invasive front of cancer tissues, show an increased ability to migrate and metastasize, and show loss of epithelial integrity and specialization, a phenotype known as epithelial to mesenchymal transition (EMT) [10, 17]. Cells at the invasive fronts of colorectal cancer show reduced expression of E-cadherin, lose their cell–cell adhesions, and aggressively invade the stroma [18–21]. In colorectal cancer, overexpression of actinin-4 and EMT are observed in cells at the invasive front. We generated colorectal cancer cells (DLD1-TetOff-*ACTN4*) that express N-terminal HA-tagged actinin-4 under control of the tetracycline regulatory system and confirmed the distribution of exogenous actinin-4 with immunocytochemistry. When exogenous actinin-4 was overexpressed in DLD1-TetOff-*ACTN4* cells, filopodia and lamellipodia, which are involved in cell migration, were predominantly observed on the cell surface compared with before overexpression. DLD1-TetOff-*ACTN4* cells are highly motile in a cell motility assay, and animal experiments to confirm the metastatic ability revealed a significant increase in lymph node metastases compared with control cells [22]. On the other hand, an siRNA-mediated decreased in actinin-4 protein in a colon cancer cell line (SW480) reduces the cellular protrusions that are associated with cancer invasion [23].

Similar phenomena are observed in pancreatic cancer. Overexpression of actinin-4 is observed in invasive ductal adenocarcinoma of the pancreas, and such patients have a worse prognosis for overall survival than patients with weak actinin-4 expression [24]. In addition, actinin-4 is mainly observed at the invadepodia of cells from a pancreatic cancer cell line [25]. When actinin-4 is reduced in the pancreatic cancer cell line, BxPC3-KD-ACTN4, with *ACTN4* siRNA, the invasive ability in the invasion assay is decreased [23]. Transplantation of pancreatic cancer cells with siRNA-mediated reduction of *ACTN4* expression into the pancreas of mice revealed no destructive invasion into the pancreas compared with control cells. Patients with ovarian cancer with overexpression of actinin-4 show similar results, including a worse outcome than patients without overexpression [26].

Interestingly, Angrwal et al. recently showed that actinin-4 interacts with murine double minute 2 homolog (MDM2) binding protein (MTBP) [27, 28]. MDM2 is a major negative regulator of the tumor suppressor, p53, but also has p53-independent roles in tumorigenesis [29]. Iwakuma et al. reported that MTBP suppresses tumor metastasis and revealed an endogenous protein–protein interaction between actinin-4 and MTBP [28]. They showed that constitutive overexpression of actinin-4 in two different osteosarcoma cell lines, SaO2-LM7 (p53 null) and U2OS (p53 wild-type), increases the migration potential in both cell lines as expected. However, concomitant overexpression of MTBP significantly decreases the potential for cell migration that is mediated by overexpression of actinin-4 in both cell lines [27, 28]. Thus, MTBP inhibits cell migration that is mediated by overexpression of actinin-4 independent of p53.

**Fig. 2:**
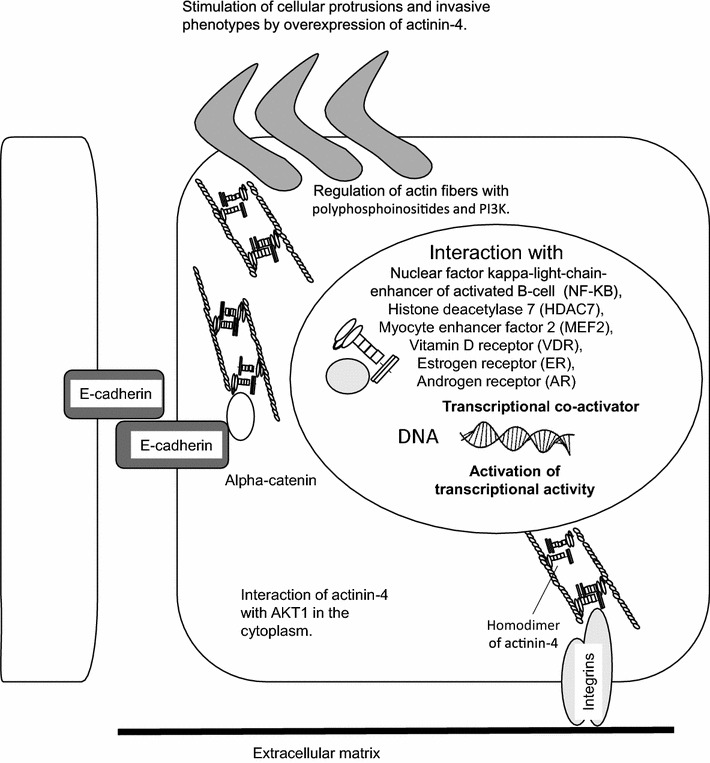
Cellular distribution of protein complexes that include actinin-4. The protein complexes that include actinin-4 and some binding partners are located in the cytoplasm and nucleus, and include integrins [14], alpha catenins [14], polyphosphoinositides [76], phosphoinositide 3-kinase (PI3K) [77], v-akt murine thymoma viral oncogene homolog 1 (AKT1) [78], nuclear factor kappa-light-chain-enhancer of activated B-cell (NF-κB) [75], histone deacetylase 7 (HDAC7) [65, 70], myocyte enhancer factor 2 (MEF2) [65, 79], vitamin D receptor (VDR) [15, 70], estrogen receptor (ER) [79], and androgen receptor [80] (AR).

In addition, actinin-4 is overexpressed in colorectal cancer [22, 23], pancreatic cancer [24, 25], ovarian cancer [26], osteosarcoma [27, 28], lung cancer [30–33], oral squamous cell carcinoma [34], salivary gland carcinoma [35], bladder cancer, breast cancer [36, 37], and esophageal cancer [38]. Reports describing overexpression of actinin-4 in association with metastasis and malignant phenotypes in cancers are summarized in Table 1. The biological function and binding partners of actinin-4 that are associated with cell invasion are shown in Fig. 2.

**Table 1:** Representative reports describing the importance of actinin-4 in malignant tumors

Type of malignant tumor	Observations
Brain tumors	1. Correlation between histological grade and protein expression of actinin-4 in gliomas [81] 2. Association of actinin-4 with cell migration in gliomas [82] 3. Overexpression of actinin-4 in high-grade astrocytomas [83]
Head and neck cancer	4. Positive correlation between *ACTN4* amplification and the histological grade of salivary gland carcinomas. The importance of *ACTN4* amplification as a prognostic biomarker in salivary gland carcinomas [35] 5. Positive correlation between invasive classification of oral squamous cell carcinoma and protein expression of actinin-4 [34] 6. Correlation between histological grade and protein expression in thyroid cancer [84]
Lung cancer	7. Utility of *ACTN4* amplification as a prognostic biomarker for stage I adenocarcinoma of the lung [50] 8. Overexpression of actinin-4 mRNA in NSCLC [31] 9. Identification of a splice variant of actinin-4 in SCLC as a cancer testis antigen [30]. Utility of a splice variant of actinin-4 in the lung as a prognostic biomarker for high-grade malignant neuroendocrine tumors [33] 10. Expression of actinin-4 in blood samples of patients with NSCLC and utility as a diagnostic biomarker for NSCLC [85]
Breast cancer	11. Identification of actinin-4 as a novel actin-bundling protein, and utility of actinin-4 as a prognostic biomarker for invasive ductal breast cancer [13] 12. Summary of actinin-4 as a translational coactivator in breast cancer [15, 65] 13. Identification of protein–protein interactions between estrogen receptors and actinin-4 [70]
Esophageal cancer	14. Overexpression of actinin-4 according to clinical stage in esophageal cancer [74]
Pancreatic cancer	15. First evidence of *ACTN4* amplification in cancer. Identification of actinin-4 overexpression in patients with invasive ductal adenocarcinoma of the pancreas with poor prognosis [24] 16. Clinical utility of *ACTN4* amplification as a predictive biomarker for chemoradiotherapy in LAPC [57] 17. Association of actinin-4 with invadopodia in pancreatic cancer [25]
Colorectal cancer	18. Identification of overexpression of actinin-4 in areas of EMT in colorectal cancer [22] 19. Involvement of actinin-4 in the formation of cellular protrusions that are associated with invasion and migration [23]
Ovarian cancer	20. Identification of actinin-4 overexpression in ovarian cancer, and correlation between actinin-4 overexpression and overall survival in patients with ovarian cancer [26] 21. Utility of *ACTN4* amplification as a prognostic biomarker in ovarian cancer [48] 22. Accumulation of *ACTN4* amplification in high-grade clear cell carcinoma of ovarian cancer [49] 23. Identification of *ACTN4* amplification in fallopian tube carcinomas [86]
Bladder cancer	24. Reduced invasive ability with *ACTN4* siRNA in bladder cancer cell lines [36] 25. Correlation between histological grade in bladder cancer and actinin-4 protein expression [37]
Prostate cancer	26. Protein complex that includes actinin-4 and androgen receptor in the nucleus. Actinin-4 protein expression is reduced in the nucleus of high-grade prostate cancer [80]
Melanoma	27. Association of actinin-4 with amoeboid-type invasiveness of melanoma cells [87]
Leukemia	28. Identification of the fusion gene *MLL*-*ACTN4* in adult CD10-negative B-cell precursor acute lymphoblastic leukemia [88, 89]
Osteosarcoma	29. Protein–protein interactions between MTBP and actinin-4 in osteosarcoma [27, 28]

## Amplification of *ACTN4* in cancer and clinical utility as a biomarker for decisions regarding the therapeutic strategy

Although overexpression of actinin-4 protein has been reported in several types of cancers, the cause of overexpression is not clearly understood. If overexpression is due to a genetic alteration in *ACTN4*, an oncogene may be actively associated with cancer invasion and metastasis. *ACTN4* is located on chromosome 19q13, and amplification of the 19q13.1 locus has been reported frequently in several cancers including pancreatic and ovarian cancers [39–47]. We used specific fluorescence in situ hybridization (FISH) of *ACTN4* to investigate *ACTN4* amplifications in patients with pancreatic cancer [24]. *ACTN4* amplifications occurred in 38% of patients with invasive ductal adenocarcinoma of the pancreas with protein overexpression of actinin-4 [24]. Patients with ovarian cancer and *ACTN4* amplification have been observed, and patients with stage III and IV disease with gene amplification show significantly worse overall survival than patients without gene amplification [48]. The status of gene amplification may more accurately predict the outcome of patients with stage III and IV ovarian cancer than immunohistochemical analysis with the anti-actinin-4 antibody. In addition, positive statistical significance between *ACTN4* amplification and the efficacy of post-operative chemotherapy was seen in patients with stage III and IV ovarian cancer [48, 49].

The clinical benefits of *ACTN4* amplification as a prognostic factor are also observed in stage I adenocarcinoma of the lung and salivary gland carcinoma, and *ACTN4* amplification is a stricter prognostic biomarker than immunohistochemistry for overall survival in these patients.

Although the clinical benefit of adjuvant chemotherapy in patients with non-small cell lung cancer (NSCLC) who have undergone complete surgical resection has been observed in stage II–IIIA in some prospective clinical trials, the benefit has not been seen in stage I NSCLC [50–52]. If patients with stage I adenocarcinoma of the lung with potential metastasis can be identified with *ACTN4* amplification of surgical specimens, adjuvant chemotherapy for such patients may have a clinical benefit in terms of patient selection.

Compared to prognostic biomarkers, predictive biomarkers to select a specific treatment strategy by evaluating the metastasis ability are urgently needed. For example, local treatment such as surgery and radiotherapy could be effective in patients without distant metastases. However, local therapies are not sufficiently effective for metastatic lesions. Patients with micrometastasis, which cannot be detected with imaging, should not undergo local therapy. Treatment options for locally advanced pancreatic cancer (LAPC) include chemotherapy alone, induction chemotherapy followed by chemoradiotherapy (CRT), or definitive CRT. Numerous randomized trials have been performed to compare the survival benefit of chemotherapy alone and CRT for LAPC [53, 54]. Results have been contradictory, and the most effective treatment has not been defined for patients with LAPC [55, 56]. Radiotherapy involving the primary site does not have sufficiently high impact for patients with occult distant metastasis, because radiotherapy does not treat distant metastatic lesions. However, imaging technology to accurately detect extremely small micrometastatic lesions has not been developed. Therefore, identification of biomarkers that can accurately evaluate the metastatic potential of biopsy samples from patients with LAPC will be very important for deciding the best personalized therapeutic strategy.

We used biopsy specimens and FISH analysis to retrospectively investigate the *ACTN4* copy number in patients with LAPC who underwent chemotherapy or CRT [57]. In such patients who underwent CRT, those with a normal *ACTN4* copy number showed a better prognosis for overall survival than patients with an increased *ACTN4* copy number. However, in patients who underwent chemotherapy, no statistically significant difference was observed between increased and normal *ACTN4* copy numbers. Thus, *ACTN4* may be a potential biomarker for metastatic ability and for predicting the effectiveness of CRT in LAPC [57].

## Specific expression of an alternative splice variant of *ACTN4* in small cell lung cancer (SCLC) and mutation in focal segmental glomerular sclerosis (FSGS)

A tumor-specific alternative splice variant of *ACTN4* was found in SCLC [30]. Exon 8 of *ACTN4* is skipped in this variant, and another exon is inserted in its place (ACTN4-SpEx8), resulting in the changes N249G, A251L, and S264C in exon 8 [30]. Among normal tissues and various cancer cell lines, we observed expression of ACTN4-SpEx8 only in a SCLC cell line and normal testis. Thus, ACTN4-SpEx8 is considered a cancer testis antigen. We established a specific antibody against ACTN4-SpEx8 and observed protein expression in SCLC and large cell neuroendocrine carcinoma (LCNEC) with immunohistochemistry among pathological samples of adenocarcinoma, squamous cell carcinoma, LCNEC, carcinoid, and SCLC. Patients with SCLC and LCNEC and ACTN4-SpEx8 protein expression have a worse outcome for overall survival than patients without such expression [33]. The altered amino acids, N249G, A251L, and S264C, are very close to the mutations that are observed in familial FSGS [58, 59], which occur in exon 8 of *ACTN4* and result in the changed amino acids K255E and T259I. The three-dimensional structure of ACTN2 has been studied in detail [60], and the mutated sites are located on the surface of the separation between the CH1 and CH2 domains. For actinin to bind to actin, the three-dimensional structure of the CH1 and CH2 domains of actinin changes from a closed to an open conformation [61–63]. The substitution of amino acids in ACTN4-SpEx8 and the *ACTN4* mutation in familial FSGS may affect the conformation of these domains.

Moreover, alteration of the affinity of actinin-4 for binding to actin filaments may be an important factor in the poor prognosis of SCLC and the effacement of foot processes in the podocytes of the glomerulus in FSGS. Recently, Ehrlicher and Pollak et al. demonstrated that in FSGS, a K255E mutation in *ACTN4* changes the cellular biological properties in which increasing the affinity for actin increases cellular forces and work and decreases cellular movement. This type of mutation in this part in *ACTN4* affects actinin binding kinetics to modulate cellular dynamics and force generation, and suggests the mechanisms by which such physical defects lead to human diseases [64].

## The role of actinin-4 as a transcriptional coactivator in cancer

Aberrant transcripts that fail to regulate the expression of mRNA are a cause of cancer development. Transcription of mRNA is strictly regulated in normal cells. Nuclear localization of actinin-4 is frequently observed in breast cancer [13], ovarian cancer [26], and SCLC [30] cells. However, the biological role of this nuclear localization is not clear, although a novel function other than cancer invasion is likely.

Early observation of actinin-4 as a transcriptional coactivator began with a report of protein–protein interactions among actinin-4, class II histone deacetylases, and myocyte enhancer factor 2s (MEF2s). A protein complex containing these three proteins increases the transcriptional activity of MEF2s. Chakraborty and Kao’s group provided the first evidence that actinin-4 plays a role as a transcriptional coactivator [65]. Moreover, they reported the important roles of actinin-4 in breast cancer cell nuclei. Nuclear hormone receptors including the vitamin D receptor and steroid hormone receptors such as the estrogen receptor (ER) are ligand-activated transcription factors that control homeostasis, cell differentiation, proliferation, and development [66–68]. In particular, the ER plays a very important role in the development of breast cancer, and Tamoxifen, a competitive inhibitor of ERs, is used as a molecular targeted drug in ER-positive patients [69]. Recently, Kao’s group also reported that estradiol (E2) promotes recruitment of actinin-4 to the promoter of *pS2*, an ER target gene in the ER-positive breast cancer cell line, MCF7 [70, 71]. The fact that actinin-4 regulates ER-alpha-mediated transcriptional activation suggests that actinin-4 may play a role in E2-mediated regulation of breast cancer cell proliferation. In fact, decreased actinin-4 protein expression due to siRNA in MCF7 cells significantly reduces E2-mediated induction of ER-alpha target genes and abolishes estrogen-mediated proliferation of cancer cells [70]. In addition, actinin-4 and ER interact [71], suggesting that actinin-4 functions as a transcriptional co-activator with ER-alpha in some subtypes of breast cancer [15].

Nuclear factor-kappa B (NF-κB) is a transcription factor that regulates cell proliferation, the immune response, cell differentiation, and apoptosis by controlling the expression of mRNA for genes encoding inflammatory cytokines, chemokines, and adhesion molecules [72, 73]. Babakov et al. reported that actinin-4 and NF-κB change their cellular localization from the cytoplasm to the nucleus when actin fibers are disrupted by cytochalasin D. The interaction between actinin-4 and NF-κB was demonstrated with immunoprecipitation following epidermal growth factor or tumor necrosis factor-alpha (TNF-alpha) stimulation [74]. Zaho et al. also clearly demonstrated that actinin-4 expression is essential for the transcriptional activity of NF-κB in the presence of TNF-alpha [75]. The NF-κB and TNF-alpha pathway is important for cancer development, during which actinin-4 may play an important role in regulating transcription events through the NF-κB pathway. The biological functions of actinin-4 as a transcriptional co-activator are summarized in Fig. 2.

Although the nuclear localization of actinin-4 was reported in breast cancer, ovarian cancer, prostate cancer, and SCLC, a correlation between the localization of actinin-4 and clinical findings of patients with cancer is not clearly understood. The biological mechanisms of the translocation of actinin-4 to the nucleus from the cytoplasm should be clarified for innovative drug development for actinin-4.

## Conclusion and future perspective

Here we described the biological roles of actinin-4, which is closely associated with cancer invasion and cell motility. Overexpression of actinin-4 protein and *ACTN4* amplification are biomarkers for evaluating the potential metastatic ability in an individual patient with cancer, and actinin-4 expression may be useful for selecting the optimal therapy for patients. In particular, predicting late metastasis after surgery is an important clinical issue. To utilize actinin-4 as a biomarker in the clinical setting, prospective clinical trials should be done.

Recent studies of actinin-4 demonstrated not only a role in cancer invasion, but also its biological role as a transcriptional co-activator. Actinin-4 is aggressively involved in the tumorigenesis of breast cancer, and this concept is attracting a lot of attention. The localization of actinin-4 in the nucleus is very interesting in terms of tumorigenesis.

Targeted therapy for actinin-4 has not been developed. Recently, the three-dimensional structure of actinin-2 has been reported [60]. Using this information, we hope that a drug for molecular targeted therapy for actinin-4 will be developed. Although these proteins have distinct physiological and cellular functions, actinin-2 and actinin-4 share 80% similarity in amino acid sequence. Basic studies of the biology of actinin-4 have the potential to overcome human diseases.

## Abbreviations

ABD: actin-binding domain
ACTNs: alpha-actinins
CaM: calmodulin
CH: calponin homology
CRT: chemoradiotherapy
EMT: epithelial to mesenchymal transition
ER: estrogen receptor
FISH: fluorescence in situ hybridization
FSGS: focal segmental glomerular sclerosis
LCNEC: large cell neuroendocrine carcinoma
LAPC: locally advanced pancreatic cancer
MDM2: murine double minute 2 homolog
MTBP: murine double minute binding protein
MEF2: myocyte enhancer factor 2
NSCLC: non-small cell lung cancer
NF-κB: nuclear factor-kappa B
SCLC: small cell lung cancer
ACTN4-SpEx8: splice variant of ACTN4
TNF-alpha: tumor necrosis factor-alpha
